# Supplementary material for: Chronic Morphine Treatment and Antiretroviral Therapy Exacerbate HIV-Distal Sensory Peripheral Neuropathy and Induce Distinct Microbial Alterations in the HIV Tg26 Mouse Model
Source: Int J Mol Sci. 2024 Jan 26;25(3):1569. doi: 10.3390/ijms25031569 (PMC10855564; doi:10.3390/ijms25031569)
Supplement: Supplementary file 1 [file ijms-25-01569-s001.zip › ijms-2783175-supplementary.pdf]

International Journal of
Molecular Sciences

MDPI

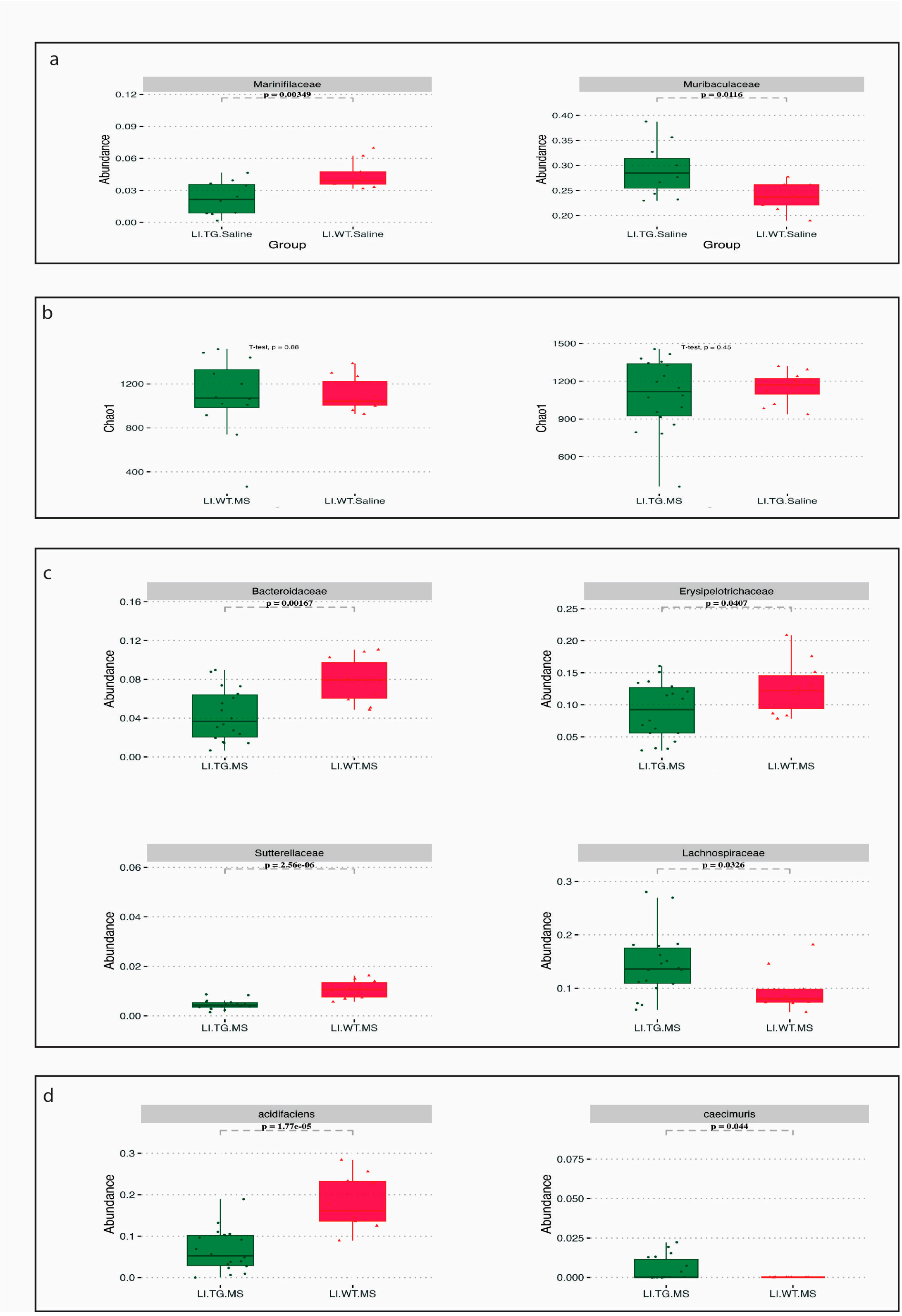

Figure S1. Distinct bacterial community are observed between WT and Tg26 mice before and after morphine treatment.

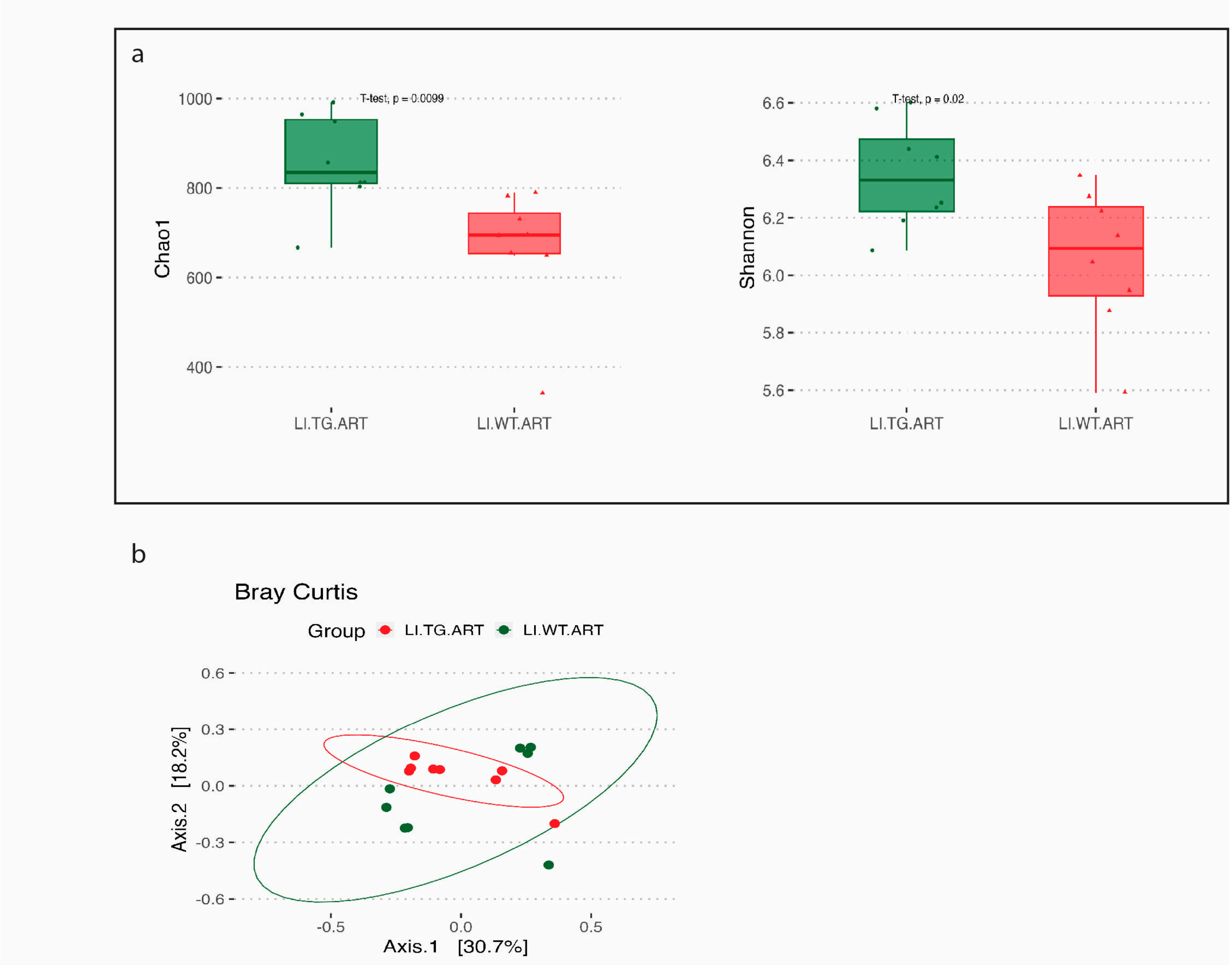

Figure S2. ART treatment modulates the gut microbiome in WT and Tg26 mice.
